# The relationship between diabetic retinopathy and diabetic nephropathy in type 2 diabetes

**DOI:** 10.3389/fendo.2024.1292412

**Published:** 2024-01-26

**Authors:** Qian Wang, Haimei Cheng, Shuangshuang Jiang, Li Zhang, Xiaomin Liu, Pu Chen, Jiaona Liu, Ying Li, Xiaocui Liu, Liqiang Wang, Zhaohui Li, Guangyan Cai, Xiangmei Chen, Zheyi Dong

**Affiliations:** ^1^Department of Nephrology, First Medical Center of Chinese PLA General Hospital, Nephrology Institute of the Chinese People’s Liberation Army, State Key Laboratory of Kidney Diseases, National Clinical Research Center for Kidney Diseases, Beijing Key Laboratory of Kidney Disease Research, Beijing, China; ^2^Senior Department of Ophthalmology, The Third Medical Center of PLA General Hospital, Beijing, China

**Keywords:** diabetic nephropathy, non-diabetic renal disease, diabetic retinopathy, diabetes mellitus, KW nodules

## Abstract

**Context:**

Diabetic retinopathy (DR) and diabetic nephropathy (DN), are major microvascular complications of diabetes. DR is an important predictor of DN, but the relationship between the severity of DR and the pathological severity of diabetic glomerulopathy remains unclear.

**Objective:**

To investigate the relationship between severity of diabetic retinopathy (DR) and histological changes and clinical indicators of diabetic nephropathy (DN) in patients with type 2 diabetes mellitus (T2DM)

**Methods:**

Patients with T2DM (n=272) who underwent a renal biopsy were eligible. Severity of DR was classified as non-diabetic retinopathy, non-proliferative retinopathy, and proliferative retinopathy (PDR). Relationship between DN and DR and the diagnostic efficacy of DR for DN were explored.

**Results:**

DN had a higher prevalence of DR (86.4%) and DR was more severe. The sensitivity and specificity of DR in DN were 86.4% and 78.8%, while PDR was 26.4% and 98.5%, respectively. In DN patients, the severity of glomerular lesions (p=0.001) and prevalence of KW nodules (p<0.001) significantly increased with increasing severity of DR. The presence of KW nodules, lower hemoglobin levels, and younger age were independent risk factors associated with more severe DR in patients with DN.

**Conclusion:**

DR was a good predictor of DN. In DN patients, the severity of DR was associated with glomerular injury, and presence of KW nodules, lower hemoglobin levels and younger age were independent risk factors associated with more severe DR.

**Trial registration:**

ClinicalTrails.gov, NCT03865914.

## Introduction

The global prevalence of diabetic nephropathy (DN) has risen substantially over the past few decades, mostly driven by an increase in the prevalence of type 2 diabetes mellitus (T2DM). The incidence of DN in patients with diabetes is 35-40% (1, 2), with diabetes and DN representing major causes of end stage renal disease (ESRD) (3). The early symptoms of DN are not easy to detect, but gross proteinuria identifies patients at risk of progression to ESRD (4). Many patients eventually need maintenance dialysis or kidney transplantation, resulting in a considerable clinical and economic burden (5).

Diabetic retinopathy (DR) and DN are major microvascular complications of diabetes. Both DR and DN have an insidious onset and a gradual progression to irreversible damage. The incidence of DR in patients with diabetes is 34.6%, and the incidence of proliferative diabetic retinopathy (PDR) is 7%. Globally, PDR is the most frequent cause of new cases of blindness (6). Early diagnosis and treatment can delay the occurrence and progression of DN and DR, and improve prognosis in patients with diabetes.

The current gold standard for diagnosing DN is renal pathology; however, the approach is invasive, which limits its application. Retinal blood vessels can be observed using non-invasive imaging. This procedure may inform on the development of other microvascular complications of diabetes, including DN. Accordingly, some evidence suggests that the retinal vascular fractal dimension is a shared biomarker of diabetic microvasculopathy, indicating a possible common pathogenic pathway (7). In patients with type 1 diabetes mellitus (T1DM), retinal vessel diameters were associated with renal structural changes. Specifically, baseline central retinal arteriolar diameter was correlated with changes in the glomerulopathy index, and central retinal venule diameter was correlated with changes in the mesangial matrix volume fraction (8). The pathological manifestations of T1DM without proteinuria, including glomerular basement membrane (GBM) thickness and mesangial matrix volume fraction, increased with increasing severity of DR (9).

DR is classified as nonproliferative and proliferative, defined by the absence or presence of neovascularization and retinal changes. Pathological classification of DN evaluates diabetic glomerular lesions, tubulointerstitial injury, vascular lesions, and non-diabetic glomerular lesions (10). Studies investigating the correlation between DR and the clinical manifestations of DN are limited. One report demonstrated that a greater fraction of the glomerulus was occupied by the mesangium (mesangial fractional volume [Vv (Mes/glom]) in patients with T2DM and DR compared to no DR (11). A literature search showed no reports describing the association between severity of DR and diabetic glomerular lesions in T2DM. The objective of this study was to investigate the relationship between severity of DR and histological changes and clinical indicators of DN in patients with T2DM. We hypothesized that the severity of DR is associated with microvascular lesions within glomeruli. Findings are expected to inform diagnostic clinical prediction tools and management of patients with DM.

## Materials and methods

### Study population

**Figure 1:**
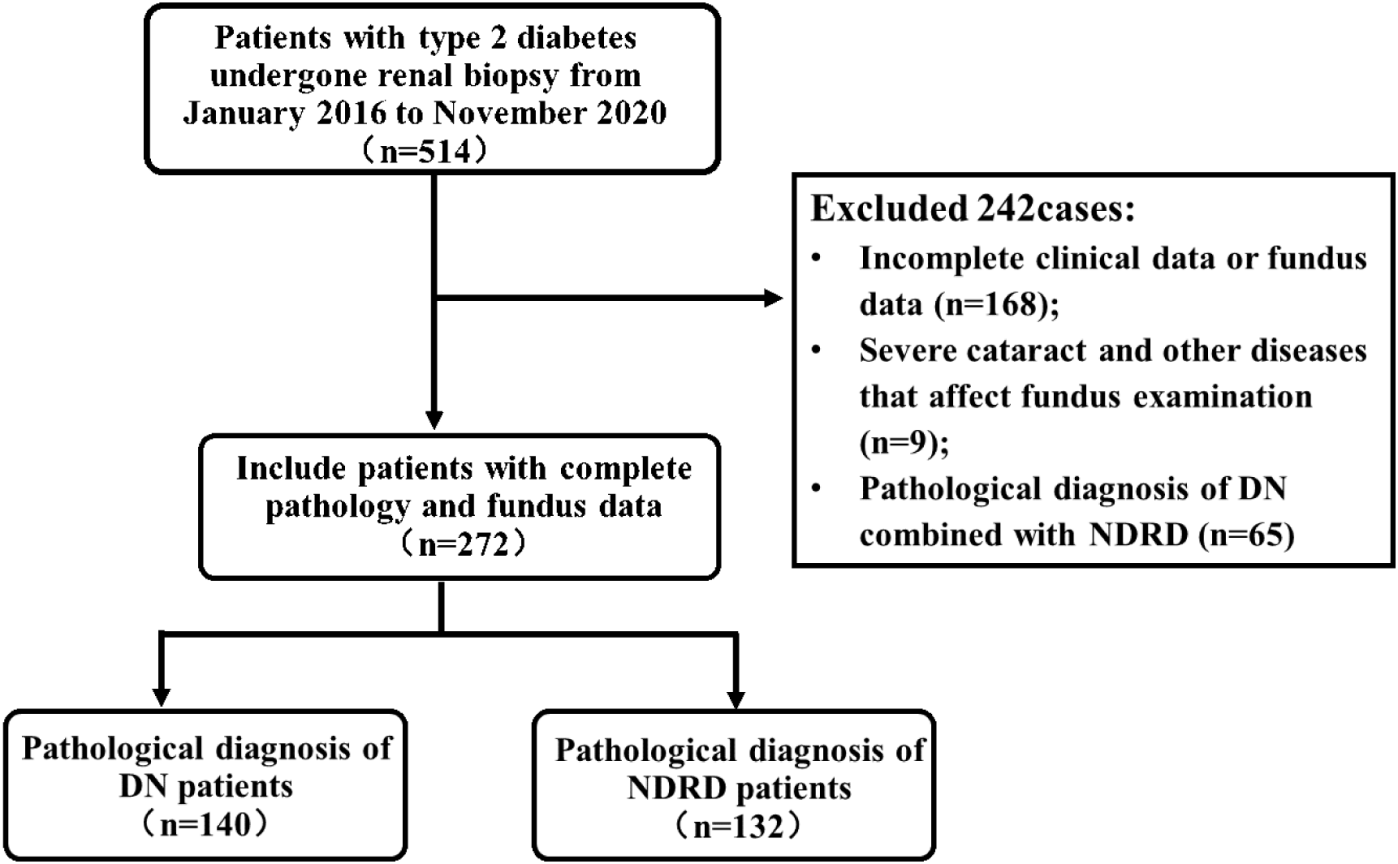
Flow chart of patient selection.

A total of 272 patients with T2DM who underwent a renal biopsy at the Chinese People’s Liberation Army General Hospital (Beijing, China) between January 2016 and November 2020 were eligible for this study (**Figure 1**). All patients provided written informed consent for a renal biopsy. Inclusion criteria were: 1) male or female, aged >18 years; 2) biopsy-proven renal lesion; and 3) underwent a fundus examination. Exclusion criteria were: 1) incomplete data or lack of a fundus examination; 2) severe cataracts or other diseases that affect a fundus examination; or 3) pathological diagnosis was DN combined with non-diabetic renal disease (NDRD). The study was approved by the Ethics Committee of the Chinese People’s Liberation Army General Hospital (No. S2017-133-01). All patients provided written informed consent. Included patients were categorized as DN, defined as patients with T2DM and DN only, or NDRD, defined as patients with T2DM and NDRD only.

### Demographic, clinical and laboratory information

Patients’ demographic and clinical characteristics were recorded, including: gender, age, medical history of DM, family history, body mass index (BMI), presence/absence of hypertension, systolic blood pressure (SBP), diastolic blood pressure (DBP), mean arterial pressure, and presence/absence of retinopathy. Laboratory parameters, including hemoglobin, serum creatinine, estimated glomerular filtration rate (eGFR, calculated by the CKD-EPI formula), serum albumin, glycated hemoglobin, 24-h urine protein and presence/absence of glomerular hematuria, were collected at the time of renal biopsy.

### Renal biopsy and pathological evaluation

All patients underwent a renal biopsy after they signed the informed consent form. Renal biopsies were performed by an experienced physician. All renal biopsy specimens were reviewed independently by two pathologists. Disagreements were solved by discussion until consensus was reached. DN was diagnosed based on the following criteria: mesangial proliferation, diffuse capillary glomerulosclerosis, presence or absence of Kimmelstiel-Wilson (KW) nodules, diffuse thickening of the GBM, and exudative injury such as fibrous cap or/and hyaline thrombi. Pathological findings were evaluated according to the Pathologic Classification of Diabetic Nephropathy (10).

### Measurement, evaluation, and diagnosis of DR

DR was diagnosed based on the results of fundus photography and/or fluorescein imaging. 45° mydriatic fundus photographs in 7 standard fields were taken with a Japanese KOWA fundus camera (VX-20; nonmyd 7). 45° fundus fluorescein angiography images were taken with the German Heidelberg Laser Ophthalmological Diagnostic Apparatus (SPECTRALIS HRA). Fundus photography and fluorescein imaging were performed by professional ophthalmologists who had undergone strict training to ensure consistency across images. Severity of DR was classified into 5 categories according to the International Clinical DR Severity Scale: “no retinopathy”, “mild non-proliferative DR”, “moderate non-proliferative DR”, “severe non-proliferative DR”, or “proliferative DR” (12). The diagnosis and evaluation of DR was performed by two ophthalmologists. Disagreements were solved by discussion with a senior ophthalmologist. In patients where DR severity was inconsistent in the two eyes, diagnosis and evaluation were based on the worst eye.

### Statistical analysis

All statistical analyses were performed using SPSS version 25.0 (SPSS Inc., Chicago, IL, USA). Continuous variables with a normal distribution were expressed as mean ± standard deviation (SD), and differences between groups were compared using analysis of variance or t−test. Multiple comparisons adopted the least significant difference method. Continuous variables with a non-normal distribution were expressed as median (Q1, Q3), and differences between groups were compared using the Kruskal–Wallis test or Mann–Whitney U−test. Qualitative data were expressed as absolute values and percentages and were compared using the Chi−square test. Clinical parameters that were significant at the 0.05 level on univariate logistic regression analysis were assessed to evaluate their contributions to severity of retinopathy. Diagnostic efficacy of DR for DN was estimated by calculating the sensitivity, specificity, positive predictive value, and negative predictive value. All P values were two-sided, with values <0.05 considered statistically significant.

## Results

### Pathology on renal biopsy

On renal biopsy, 140 patients (51.5%) were histologically diagnosed with DN, and132 patients (48.5%) were histologically diagnosed with NDRD. Among the patients diagnosed with NDRD, membranous glomerulonephritis (51 patients, 38.6%) was the most common glomerular NDRD, 33 (25.0%) patients were diagnosed with immunoglobulin A nephropathy, 19 patients (14.4%) were diagnosed with focal segmental glomerulosclerosis, and 7 patients (5.3%) were diagnosed with interstitial nephritis (**Table 1**).

**Table 1:** Pathology on renal biopsy.

Kidney pathology	n (%)
DN	140
NDRD	132
Membranous nephropathy	51 (38.6%)
IgA nephropathy	33 (25.0%)
Focal segmental glomerulosclerosis	19 (14.4%)
Interstitial nephritis	7 (5.3%)
Minimal change glomerulopathy	4 (3.0%)
hypertensive renal damage	3(2.3%)
Membranoproliferative glomerulonephritis	3 (2.3%)
Focal glomerulosclerosis	3 (2.3%)
Obesity related glomerulopathy	2 (1.5%)
Purpuric nephritis	2 (1.5%)
Mesangial proliferative glomerulonephritis	2 (1.5%)
Endothelial cell disease	1 (0.8%)
C3 nephropathy	1 (0.8%)

### Clinical characteristics in patients with DN or NDRD

Compared to patients with NDRD, patients with DN had a significantly longer duration of DM [156 (75, 204) vs. 48 (12, 96)months; p<0.001], significantly higher SBP [142 (135,160) vs. 132 (120,145) mmHg; p<0.001] and mean arterial pressure [104.5 ± 13.5 vs. 99.4 ± 13.7 mmHg; p=0.002], significantly higher HbA1c [7.0 (6.3, 8.0) vs. 6.6 (6.0,7.2)%; p<0.001], urea nitrogen [9.9 (6.8,12.9) vs. 6.6 (5.1, 8.3)µmol/L; p,0.001], serum creatinine[159.8 (102.3, 224.1) vs. 94.3 (77.7,125.4) µmol/L; p<0.001] and urine protein [4.3(2.2,6.4) vs. 2.6 (1.0, 4.9)g/24h; p<0.001] levels, and a significantly higher prevalence of DR[86.4% vs. 21.2%; p<0.001]. Compared to patients with NDRD, patients with DN had a significantly lower BMI[26.1 (23.9, 28.6) vs. 26.9 (25.2, 29.1) kg/m^2^; p=0.013] significantly lower hemoglobin [113.4 ± 20.2 vs. 133.3 ± 20.9 g/L; p<0.001], total cholesterol [4.5(3.7, 5.4) vs. 5.0 (4.0, 6.1) mmol/L; p=0.006], triglyceride [1.8 (1.2, 2.5) vs. 2.1 (1.5, 3.2)mmol/L; p=0.001] and low density lipoprotein [2.7 (2.0, 3.7) vs. 3.1 (2.4, 4.1)mmol/L; p=0.008]levels, and a significantly lower eGFR [42.0 (24.6,67.7) vs. 72.5 (55.1,92.2) ml/min/173m^2^; p<0.001] (**Table 2**).

**Table 2:** Clinical characteristics of patients with DN or NDRD.

Characteristic	DN (n=140)	NDRD (n=132)	P
Sex, (male %)	110 (78.6%)	102 (77.3%)	0.796
Age(years)	53 (47.0,58.8)	54 (46.0,60.0)	0.739
Duration of diabetes (month)	156 (75,204)	48 (12,96)	<0.001
SBP(mmHg)	142 (135,160)	132 (120,145)	<0.001
DBP(mmHg)	83.7 ± 12.4	82.7 ± 12.2	0.527
Mean arterial pressure(mmHg)	104.5 ± 13.5	99.4 ± 13.7	0.002
BMI (kg/m^2^)	26.1 (23.9,28.6)	26.9 (25.2,29.1)	0.013
Hemoglobin (g/L)	113.4 ± 20.2	133.3 ± 20.9	<0.001
Fasting plasma glucose(mmol/L)	6.0 (4.7,8.2)	5.6 (4.8,6.6)	0.077
HbA1c (%)	7.0 (6.3,8.0)	6.6 (6.0,7.2)	<0.001
Total cholesterol (mmol/L)	4.5 (3.7,5.4)	5.0 (4.0,6.1)	0.006
Triglyceride (mmol/L)	1.8 (1.2,2.5)	2.1 (1.5,3.2)	0.001
High density lipoprotein (mmol/L)	1.0 (0.9,1.2)	1.0 (0.8,1.2)	0.546
Low-density lipoprotein (mmol/L)	2.7 (2.0,3.7)	3.1 (2.4,4.1)	0.008
Albumin(g/l)	33.5 ± 6.2	34.5 ± 7.9	0.274
Urea nitrogen(umol/L)	9.9 (6.8,12.9)	6.6 (5.1,8.3)	<0.001
Serum creatinine(umol/L)	159.8 (102.3,224.1)	94.3 (77.7,125.4)	<0.001
Uric acid(umol/L)	381.2 ± 91.6	385.8 ± 93.8	0.685
eGFR(ml/min/173m^2^)	42.0 (24.6,67.7)	72.5 (55.1,92.2)	<0.001
Urine protein (g/24h)	4.3 (2.2,6.4)	2.6 (1.0,4.9)	<0.001
DR (%)	121 (86.4%)	28 (21.2%)	<0.001
Mild-NPDR (%)	5 (3.6%)	8 (6.1%)	0.336
Moderate-NPDR (%)	28 (20.0%)	14 (10.6%)	0.032
Severe-NPDR (%)	51(36.4%)	4 (3.0%)	<0.001
PDR (%)	37 (26.4%)	2 (1.5%)	<0.001
No-DR (%)	19 (13.6%)	104 (78.8%)	<0.001

Values were shown as median (Q1, Q3), mean ± SD, or n (%).

BMI, body mass index; DBP, diastolic blood pressure; DR, diabetic retinopathy; GFR, glomerular filtration rate; HbA1c, glycated hemoglobin; SBP, systolic blood pressure.

### Severity of DR

Compared to patients with NDRD [n=28/132 (21.2%)], patients with DN had a significantly higher prevalence of DR [n=121/140 (86.4%)] (p<0.001), and DR was more severe (**Table 2**).

### Diagnostic efficacy of DR for DN

The sensitivity and specificity of DR to detect DN were 86.4% and 78.8%, the positive predictive value was 81.2%, and the negative predictive value was 84.6%, indicating that the probability of a pathological diagnosis of DN in patients with DR is 82.7%. The sensitivity and specificity of PDR to detect DN were 26.4% and 98.5%, the positive predictive value was 94.9%, and the negative predictive value was 55.8%, indicating that the probability of a pathological diagnosis of DN in patients with PDR was 61.4% (**Table 3**).

**Table 3:** Diagnostic efficacy of DR for DN.

	Sensitivity	Specificity	Positive predictive value	Negative predictive value	Youden index	Accuracy
DR	86.4%	78.8%	81.2%	84.6%	0.652	82.7%
PDR	26.4%	98.5%	94.9%	55.8%	0.249	61.4%

### Pathological manifestations of DN according to stage of DR

Patients with DN were classified according to stage of DR, as NDR, NPDR, and PDR. The severity of glomerular lesions (p=0.001) and prevalence of KW nodules (p<0.001) significantly increased with increasing severity of DR. There were no significant differences in interstitial fibrosis and inflammatory infiltration, vascular hyalinosis, or vascular sclerosis (**Table 4**).

**Table 4:** Pathological manifestations of DN according to stage of DR .

	NDR (n=19)	NPDR (n=84)	PDR (n=37)	P
Glomerular classification, %				0.001
I	–	–	–	
II	8 (42.1%)	14 (16.7%)	0 (0%)	
III	7 (36.8%)	52 (61.9%)	22 (59.5%)	
IV	4 (21.1%)	18 (21.4%)	15 (40.5%)	
IFTA, %				0.117
1	5 (26.3%)	13 (15.5%)	1 (2.7%)	
2	7 (36.8%)	38 (45.2%)	22 (59.5%)	
3	7 (36.8%)	33 (39.3%)	14 (37.8%)	
Arteriolar hyalinosis, %				0.793
0	2 (10.5%)	3 (3.6%)	2 (5.4%)	
1	9 (47.4%)	46 (54.8%)	20 (54.1%)	
2	8 (42.1%)	35 (41.7%)	15 (40.5%)	
Arteriosclerosis, %				0.081
0	2 (10.5%)	5 (6.0%)	7 (18.9%)	
1	2 (10.5%)	26 (31.0%)	7 (18.9%)	
2	15 (78.9%)	53 (63.1%)	23 (62.2%)	
KW lesion, %	8 (42.1%)	59 (70.2%)	35 (94.6%)	<0.001

IFTA, Interstitial fibrosis and tubular atrophy.

### Clinical features of DR in patients with DN

In patients with DN and NDR, NPDR or PDR, there were significant differences in age, HbA1c, hemoglobin, albumin, total cholesterol, urea nitrogen, and urine protein (P<0.05). Compared to patients with PDR, patients with NDR or NPDR were significantly older and had significantly higher hemoglobin levels and albumin levels, and significantly lower total cholesterol levels, and patients with NDR had significantly lower urea nitrogen and urine protein levels. Compared to patients with NPDR, patients with NDR had significantly higher hemoglobin levels and significantly lower urine protein levels, and patients with PDR had significantly lower hemoglobin levels (**Table 5**).

**Table 5:** Clinical features of DN according to stage of DR.

Characteristics	No-DR (n=19)	NPDR (n=84)	PDR (n=37)	p-value
Sex(male %)	17 (89.5%)	68 (81.0%)	25 (67.6%)	0.117
Age(years)	55.2 ± 8.8*	53.8 ± 9.4*	47.2 ± 8.9	0.001
BMI (kg/m^2^)	27.6 ± 2.8	26.0 ± 3.3	25.9 ± 3.8	0.160
Duration of diabetes (month)	126.6 ± 57.5	152.6 ± 86.6	131.0 ± 74.5	0.251
SBP(mmHg)	140.0 (130.0,155.0)	143.0 (135.0,157.0)	147.0 (134.0,166.5)	0.270
DBP(mmHg)	80.0 (73.5,90.0)	82.5 (78.5,90.0)	85.0 (75.5,93.5)	0.276
Mean arterial pressure(mmHg)	100.1 (94.0,106.7)	103.3 (94.3,112.3)	106.7 (96.3,117.5)	0.342
HbA1c (%)	7.2 (6.8,8.5)	7.3 (6.5,8.2)	6.6 (5.8,7.4)	0.026
Hemoglobin (g/L)	127.0 (117.5, 149.5)*#	115.0 (101.0, 128.0)*	104.0 (93.5,120.0)#	<0.001
Albumin(g/l)	36.9 ± 5.3*	34.0 ± 5.9*	30.6 ± 6.31	0.001
Fasting plasma glucose(mmol/L)	6.4 (5.1, 7.9)	6.0 (4.7, 8.3)	5.5 (4.5, 7.2)	0.459
Total cholesterol (mmol/L)	4.1 ± 1.2*	4.5 ± 1.4*	5.2 ± 1.4	0.025
Triglyceride (mmol/L)	2.1 (1.4, 2.5)	1.7 (1.1, 2.5)	2.1 (1.4, 2.7)	0.159
High density lipoprotein (mmol/L)	0.8 (0.7,1.0)	1.0 (0.9,1.3)	1.0 (0.8,1.3)	0.251
Low-density lipoprotein (mmol/L)	2.2 (1.4,3.4)	2.5 (1.9,3.7)	3.0 (2.5,3.9)	0.064
Urea nitrogen(umol/L)	6.9 (5.9,12.9) *	9.7 (7.2,12.6)	11.4 (8.3,13.9)	0.015
Serum creatinine(umol/L)	107.7 (87.5, 226.00)	159.8 (104.3, 221.9)	182.0 (116.8, 241.0)	0.077
Uric acid(umol/L)	387.8 ± 82.6	374.7 ± 92.7	392.7 ± 94.5	0.583
eGFR(ml/min/173m^2^)	64.9 (26.5, 79.8)	42.0 (24.1, 67.8)	35.0 (25.0, 59.8)	0.086
Urine protein (g/24h)	2.1 (0.6,5.7)#*	4.1 (2.3,6.4)	5.0 (2.7,7.0)	0.004

Values were shown as median (Q1, Q3), mean ± SD, or n (%).

vs. NPDR, p<0.05; * vs. PDR, p<0.05.

### Multivariate analysis of patients with DN and different stages of DR

Multivariate analysis was performed with stage of DR as the dependent variable (NDR group=1, NPDR group=2, PDR group=3), and the factors that were significant on univariate analysis as independent variables. Findings on ordinal logistic regression (x2 = 11.245, p=0.259) satisfied the parallel regression assumption. After controlling for age, HbA1c, hemoglobin, total cholesterol and urea nitrogen levels, 24h proteinuria, and glomerular classification, presence of KW nodules (OR=5.774, 95% CI: 1.394-23.913, χ^2^ = 5.848, p=0.016), lower hemoglobin levels (OR=0.971, 95% CI: 0.946-0.996, χ^2^ = 5.020, p=0.025), and younger age (OR=0.946, 95% CI: 0.907-0.987, χ^2^ = 6.525, p=0.011) were independent risk factors associated with more severe DR (**Table 6**).

**Table 6:** Multivariate analysis of DN patients with different DR stages.

	Odds ratio	95% Confidence interval	p-value
NDR group =1			0.006
NPDR group =2			0.307
Age	0.946	0.907-0.987	0.011
Glycated hemoglobin	1.002	0.769-1.307	0.988
Hemoglobin	0.971	0.946-0.996	0.025
Urea nitrogen	1.027	0.942-1.120	0.540
Total cholesterol	1.322	0.996-1.754	0.053
Urine protein (g/24h)	0.984	0.859-1.127	0.813
K-W lesions	5.774	1.394-23.913	0.016
Glomerular classification			
Class II	1#		
Class III	0.587	0.107-3.200	0.538
Class IV	1.035	0.223-4.801	0.965

1# Reference variable.

## Discussion

Accumulating evidence suggests that DR is an important predictor of DN (13, 14). The present study showed that DR can predict DN with high sensitivity (86.4%), and PDR can predict DN with high specificity (98.5%). As DR and DN are major microvascular complications of diabetes that share similar underlying pathophysiological mechanisms, and DR is useful for diagnosing DN, the present study explored the relationship between the severity of DR and histological changes and clinical indicators of DN in patients with T2DM. Findings will further our understanding of microvascular complications of diabetes.

Retinal blood vessels can be observed on non-invasive imaging and are commonly used clinically to assist in the diagnosis of other microvascular complications of diabetes. Previous studies have investigated the relationship between DR and DN. Findings showed a higher incidence of KW nodules in patients with T2DM and DR compared to no DR (15, 16), a greater Vv (Mes/glom) in normo-microalbuminuric patients with T2DM and DR compared to no DR (11), and that the severity of renal pathology in patients with T1DM increased with the severity of DR (9). Currently, the relationship between the severity of DR and DN in patients with T2DM is unclear. Due to the common pathogenesis of advanced glycation end products (AGEs), oxidative stress, activation of polyol pathways, inflammatory factors, hemodynamic changes, and PKC (17, 18), there is likely a similarity between DR and DN disease progression.

The present study suggests that severity of DR in T2DM is associated with glomerular injury and has no correlation with renal tubular injury or vascular lesions. We speculate that DR is predictive of glomerular lesions, and interstitial changes occur as later renal pathology. The prevalence of KW nodules in patients with DN increased with severity of DR, indicating that patients with a pathological manifestation of KW nodules are more likely to have severe DR. After adjusting for age, HbA1c, hemoglobin, total cholesterol and urea nitrogen levels, 24h proteinuria, and glomerular classification, multivariate analysis showed that patients with KW nodules had a 5.77-fold higher risk of developing more severe DR compared to patients with no KW nodules. Consistent with our findings, previous reports showed that patients with T2DM, microalbuminuria and DR had established diabetic lesions on light microscopic morphometric analysis of renal biopsies and an increased mesangial volume fraction on electron microscopic morphometric analysis (11); 90% of patients with T2DM and nodular glomerular lesions had DR compared to 14% of patients with diffuse glomerulosclerosis (16); and patients with DR and concomitant microalbuminuria showed typical diabetic glomerulosclerosis and progressive renal dysfunction (11). These data suggest the severity of DR can predict the pathological changes of nephropathy, especially in patients with positive urine protein.

Our results showed that low hemoglobin level was an independent risk factor for severe DR in patients with T2DM and DN. Notably, hemoglobin was lower in patients with DN compared to NDRD, potentially due to the long course of diabetes, disorder of splanchnic innervation, or impaired release of erythropoietin (EPO) (19). Anemia is a common complication of diabetes. Consistent with our findings, previous reports identified anemia as an independent risk factor for DR (20–23). In patients with T2DM, anemia was associated with increased risk of NPDR (OR=1.75, 95% CI=1.18–2.58) and PDR (OR=3.71, 95% CI=2.23–6.18) (24), and hemoglobin was the only hematological variable that showed a significant inverse association with the severity of DR [beta-coefficient=-0.52, P value=0.003] and retinal ischemia [beta-coefficient=-0.49, P value=0.001] (23). Compared to healthy individuals, patients with diabetes have decreased red blood cell deformability, the aggregation ability of erythrocytes is increased, and the affinity of erythrocytes for oxygen is decreased, which leads to a reduction in blood oxygen carrying capacity and local hypoxia (25). Hypoxia in the retina leads to the release of inflammatory factors, vascular endothelial growth factor (VEGF), and erythropoietin, resulting in increased vascular permeability and severe DR (26). Anemia treatment may impact the severity of DR (22); therefore, we recommend that routine evaluation and treatment of anemia be performed to reduce the occurrence of microvascular complications in patients with DM.

In our study, the mean age of patients with T2DM, DN and NPDR or PDR was younger than patients with NDR, and younger age was an independent risk factor for severe DR in patients with T2DM and DN. Consistent with our findings, previous reports showed that patients with early-onset DM are at risk of developing premature DR (27–32). In patients with T2DM, the risk of DR was highest in patients who developed diabetes aged 31–45 years (OR=1.815, 95% CI=1.139–2.892]; p = 0.012) (33), patients with early-onset T2DM, defined as age of diagnosis < 40 years, had a higher prevalence of overall retinopathy after 10 years of diagnosis (27), and among patients diagnosed with diabetes before 30 years of age who developed PDR, 60% developed DN, 23% developed renal failure requiring dialysis, 24% become blind, and 10% developed atherosclerotic vascular disease (15). The tendency for patients with early-onset DM to develop microvascular complications may result from inadequate disease self-management and glycaemic control. In the present study, patients with PDR had lower HbA1c levels, possibly due to earlier detection of microangiopathy and thus tighter glycemic control. We recommend implementing strategies that help patients diagnosed with diabetes at a younger age to overcome the challenges associated with diabetes self-management by taking a whole person and behavioral health approach. This should allow patients to manage diet and medication schedules and monitor blood glucose while perusing education or a career, and raising a family.

This study was limited by sample size, despite this, to our knowledge, this is the largest published study investigating the relationship between DR and DN in patients with T2DM. Patients had a pathological diagnosis of DN on renal biopsy, and DR was diagnosed based on fundus photography and/or fluorescein imaging. Patients also had proteinuria, a decline in glomerular filtration rate, and Class II-IV glomerular lesions; therefore, injury to renal tissue varied from mild to advanced. Existing research has confirmed that progression of DN and DR are related to a decline in renal function, but have not studied the associations between the pathological characteristics of DN and DR severity. Our study was currently the largest study with fine renal biopsy indexes and DR fine grading, which filled this gap to a certain extent. Future studies will include a larger sample size and investigate the relationship between the clinical features of lesions of the ocular fundus, such as exudation and hemorrhage, and changes in renal pathology. Findings will provide insights into the pathophysiological mechanisms underlying microvascular complications of diabetes.

In patients with T2DM, DR was a good predictor of DN, and PDR predicted DN with high specificity. In patients with T2DM and DN, the severity of DR was associated with glomerular injury, but had no correlation with renal tubular injury or vascular lesions, and presence of KW nodules, lower hemoglobin levels and younger age were independent risk factors associated with more severe DR.

## Data availability statement

The original contributions presented in the study are included in the article/supplementary material. Further inquiries can be directed to the corresponding authors.

## Ethics statement

The studies involving humans were approved by the Chinese People’s Liberation Army General Hospital. The studies were conducted in accordance with the local legislation and institutional requirements. The participants provided their written informed consent to participate in this study.

## Author contributions

QW: Data curation, Formal analysis, Writing – original draft, Funding acquisition. HC: Data curation, Formal analysis, Writing – original draft. SJ: Writing – review & editing, Visualization. LZ: Writing – review & editing. XML: Data curation, Writing – review & editing. PC: Data curation, Writing – review & editing, Supervision, Visualization. JL: Data curation, Writing – review & editing, Visualization. YL: Writing – review & editing, Supervision, Validation. XCL: Writing – review & editing, Validation, Visualization. LW: Writing – review & editing, Resources, Visualization. ZL: Writing – review & editing, Resources, Visualization. GC: Writing – review & editing. XC: Conceptualization, Project administration, Writing – review & editing, Funding acquisition. ZD: Conceptualization, Writing – review & editing, Funding acquisition, Project administration.
